# Chemogenetics reveals a surprising role for GPR18 signaling in immunometabolic regulation of **β** cell activity

**DOI:** 10.1172/JCI212477

**Published:** 2026-11-16

**Authors:** Xianglin Huang, Bryan L. Roth

**Affiliations:** Department of Pharmacology, School of Medicine, University of North Carolina at Chapel Hill, Chapel Hill, North Carolina, USA.

## Abstract

Pancreatic islet macrophages are important immunometabolic regulators of β cell function, particularly in obesity-associated type 2 diabetes. Previous studies have established that pancreatic islet macrophages communicate with β cells through either the release of cytokines and growth factors, or direct cell–cell contact. In this issue of the *JCI*, Singh et al. used chemogenetics to identify a Gq/AMPK pathway that remodels sphingolipid metabolism. This pathway links macrophages to β cells through a CD36-dependent mechanism that reduces insulin secretion. Surprisingly, the orphan GPCR GPR18 was identified as the receptor that engages this pathway. These findings expand our understanding in macrophage–β cell communication and identify GPR18 as a potential therapeutic target for type 2 diabetes.

## Diabetes, GPCRs, and islet macrophages

Type 2 diabetes (T2D) is one of the most prevalent and rapidly growing chronic metabolic disorders worldwide, affecting approximately one in nine adults aged 20–79 in 2024 (1, 2). This complex disease emerges from interactions between insulin resistance, progressive β cell dysfunction, altered nutrient handling, and chronic inflammation across multiple tissues. The clinical success of glucagon-like peptide-1 receptor (GLP-1R) agonists and dual- or triple-action agonists targeting the GLP-1R/gastric inhibitory polypeptide receptor/glucagon receptor illustrates the therapeutic power of accurately targeting GPCRs in metabolic disease, especially in T2D.

Pancreatic islet macrophages have attracted increasing attention as immunometabolic regulators of β cell adaptation and failure. In obesity, these macrophages can adopt inflammatory states and release proinflammatory cytokines such as IL-1β, leading tο pancreatic β cell dysfunction and apoptosis (3, 4). Moreover, resident intra-islet macrophages can engulf insulin secretory granules from β cells through direct cell–cell interactions, but they can also compensate for this effect on β cell output by promoting β cell proliferation through platelet-derived growth factor receptor signaling (5). Many GPCRs in macrophages have also been found to mediate important communication between islet macrophages and β cells in response to metabolic stress due to obesity (Figure 1). Activation of free fatty acid receptor 4 (also known as GPR120) by fatty acids leads to IL-6 secretion and promotes insulin secretion from β cells, an effect that is impaired in obesity-associated diabetes (6). Lysophosphatidic acid receptor 5 (GPR92) similarly promotes antiinflammatory responses to improve β cell insulin secretion (7). Conversely, GPR132 shows an increased expression level in islet macrophages under high-fat diet (HFD) conditions and promotes macrophage reprogramming that impairs glucose sensing and insulin secretion. Both functions of GPR132 could be restored by a selective antagonist (8). Together, these findings position islet macrophage GPCR signaling as a potentially druggable interface between metabolic stress and β cell function.

## DREADDs as pathway-first tools for antidiabetic target discovery

Designer receptors exclusively activated by designer drugs (DREADDs) provide a way to interrogate GPCR biology with spatial and temporal control. DREADDs are engineered GPCRs that selectively activate one of several signal transduction pathways (Gq/11, Gs, Gi, G12/13, or β-arrestin) in response to a specific synthetic compound rather than endogenous ligands. Since the development of the conventional DREADD platform as a practical in vivo tool (9, 10), it has been broadly used to establish the relationships between cell-specific GPCR signaling and (patho)physiological responses and to discover and prevalidate therapeutically relevant pathways. This pathway-first strategy has been particularly informative in the research of metabolic diseases, especially in T2D, which results from imbalances across multiple cell types, including but not limited to the pancreatic cells, hepatocytes, and adipocytes (8). Early studies expressing Gq- or Gs-coupled designer receptors selectively in pancreatic β cells of transgenic mice showed that activating either pathway increased insulin release (11). Identifying pathologically relevant pathways can then direct the discovery and validation of endogenous therapeutic targets.

DREADDs were later employed to probe the effects of acute Gq activation (12) and Gi activation (13) in hepatocytes, identifying that both lead to increased hepatic glucose production and impaired glucose homeostasis. These studies resulted in the identification of V1b-vasopressin and CB1-cannabinoid receptors as potential targets in T2D. Similarly, selective Gq activation in adipocytes was found to improve glucose homeostasis and tolerance, revealing the beneficial effects of activating cysteinyl leukotriene receptor 2 (14). Given the crucial role of macrophages in obesity-associated islet remodeling, extending this approach to investigate the pathway-specific effects in these cells offers opportunities to discover unexplored GPCR targets.

## Role of myeloid Gq activation

In this issue of the *JCI*, Singh and colleagues applied this DREADD-based strategy to identify pathways involved in macrophage regulation of β cell function (15). Singh et al. first identified Gq signaling as a candidate pathway by analyzing public scRNA-seq data from cells in pancreatic islets of mice fed regular chow (RC; non-diabetic control) or an HFD (diet-induced obesity diabetic model). They found that the *Gnaq* expression level in M1 macrophages was significantly higher under the HFD than the RC condition. Similar changes in BMDMs, together with the positive correlation between *Gnaq* expression level in human PBMCs and body mass index, motivated a direct test of the functional consequences of Gq activation.

To selectively probe the role of Gq activation in macrophages, Singh et al. created a GqD mouse line with myeloid-specific expression of the Gq-coupled DREADD receptor hM3Dq (9). Administration of the selective chemogenetic ligand deschloroclozapine (DCZ) activated GqD in myeloid cells, including but not limited to macrophages, and produced changes in both glucose homeostasis and β cell insulin secretion. DCZ increased blood glucose and reduced plasma insulin levels in both RC- and HFD-fed mice, with no changes in insulin sensitivity or oral glucose tolerance. Among four insulinotropic stimuli tested, GqD stimulation impaired glucose-stimulated insulin secretion and arginine-stimulated insulin secretion in both RC- and HFD-fed mice and impaired insulin response to glyburide and GLP-1R agonist exendin-4 in HFD-fed mice. Reduced insulin secretion in response to all four insulinotropic stimuli was also observed in isolated islets from the LysM-GqDREADD mice in the presence of DCZ and was rescued with treatment of clodronate liposomes, which selectively deplete islet macrophages.

Importantly, the converse genetic experiment supported the same conclusion regarding the role of Gq. Here, myeloid-specific Gq deletion enhanced glucose-stimulated insulin secretion and therefore improved glucose clearance. Together, these complementary gain- and loss-of-function experiments elucidate the role of myeloid Gq activation in restraining insulin secretion in β cells.

## Lipid metabolism remodeling relays macrophage signals to β cells

Next, the authors showed that although in vivo activation of GqD did not alter circulating or transcript levels of typical pro- or antiinflammatory cytokines, it was associated with changes in sphingolipid metabolism (15). Lipidomic profiling revealed that ceramide and sphingomyelin species increased in both cell lysates and conditioned medium from BMDMs as well as in the whole pancreatic islet following DCZ administration, suggesting that islet macrophages actively metabolized and secreted sphingolipids, which could then affect the neighboring β cells. The Gq-triggered regulation of lipid metabolism occurred through the activation of AMPK, and the Gq-AMPK activation did not affect de novo sphingolipid synthesis but instead regulated the recycling of preexisting sphingolipids to ceramide, remodeling the local composition of sphingolipid intermediates.

The local sphingolipid intermediates then relayed the signals through a mechanism involving the lipid transport protein CD36, PKCζ, AKT, and glycogen synthase kinase 3 beta (GSK3β) (Figure 1). Ultimately, reduced GSK3β Ser9 phosphorylation disinhibited its kinase activity in β cells, leading to effects such as the destabilization of core transcription factors and therefore reduced secretory competence. Pretreatment with the CD36 inhibitor sulfosuccinimidyl oleate restored the insulin secretion from INS-1 β cells exposed to conditioned media from GqD-activated macrophages, supporting the CD36-dependent model. The conceptual advance is that macrophages can also influence β cells through changes in the local lipid environment, in addition to cytokine release or direct contact.

## GPR18 provides an endogenous entry point

Having identified the Gq/AMPK/sphingolipid/CD36/PKCζ/AKT/GSK3β axis connecting macrophages and β cells, Singh et al. next searched for an endogenous macrophage GPCR capable of engaging it (15). Murine macrophage scRNA-seq data and qPCR validation in BMDMs and isolated islets identified GPR18 as a candidate upstream regulator. GPR18 expression level also correlated positively with body weight and blood glucose level. Stimulation with the GPR18 agonist *N*-arachidonoyl glycine successfully reproduced the key effects of chemogenetic Gq activation: reduced insulin secretion in response to glucose, arginine, and glyburide; accumulation of sphingolipid species; increased AMPK phosphorylation; and reduced AKT phosphorylation. Perturbing the axis using knockdown of GPR18 or AMPK using siRNA, myeloid Gq knockout, or treatment with clodronate liposomes prevented GPR18-induced impairment of insulin secretion from β cells, demonstrating the validity and importance of the GPR18/Gq/AMPK/sphingolipid axis in islet macrophage–β cell signaling (Figure 1). This pathway was also found to be conserved in human immune cells and islets. Importantly, these findings show how chemogenetics can be employed to identify candidate druggable targets — in this case, GPR18.

## From mechanistic insight to therapeutic opportunities

Several questions remain to be resolved before the pathway Singh et al. described can be translated into therapeutic benefits. How is GPR18 engaged during disease progression: does it occur via altered ligand availability or increased receptor expression? How do individual sphingolipid species contribute to β cell dysfunction? Emerging work by the same research group has suggested that distinct macrophage pathways provide additional regulation of insulin secretion and sensitivity, thereby revealing a largely unexplored GPCR signaling map in islet macrophages (16). Together, the identification of the GPR18/Gq/AMPK/sphingolipid pathway expands our understanding of how islet macrophages affect endocrine function and provides exciting opportunities to selectively interrupt pathologic macrophage–β cell communication without perturbing beneficial macrophage functions.

## Manuscript information

### AI disclosure.

The authors used ChatGPT-5.6 Sol (OpenAI) to improve the readability and clarity of the text (September 12, 2026).

### Conflict of interest.

BLR is a member of the scientific advisory board of Epiodyne, XyloBio, SkapeBio, and ImprintBio. The University of North Carolina has licensed technologies from the Roth lab to many biotechnology and pharmaceutical companies.

## Figures and Tables

**Figure 1:**
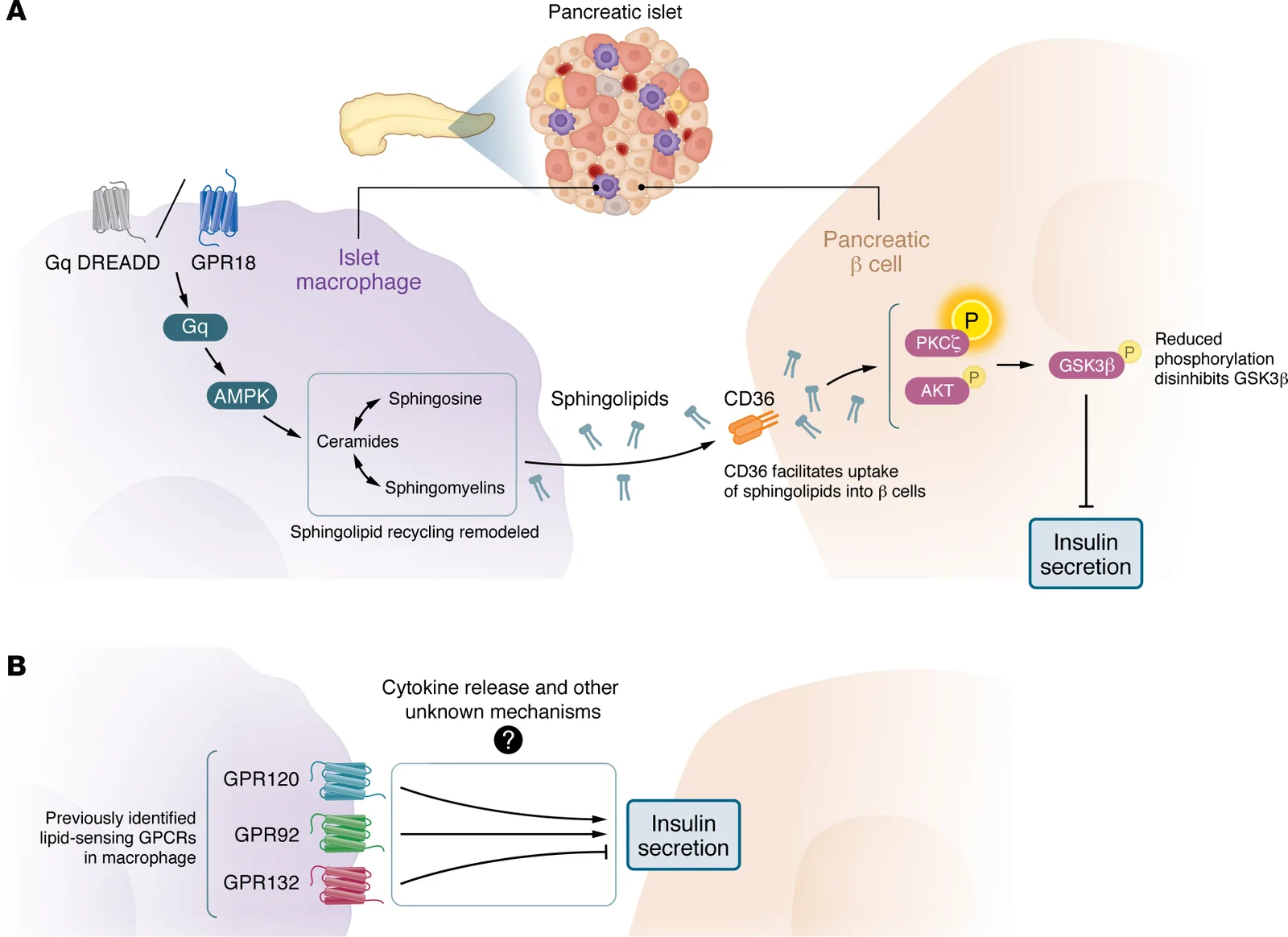
The orphan GPCR GPR18 engages a pancreatic macrophage–β cell signaling pathway that reduces insulin secretion. (**A**) Using Gq-coupled DREADD, Singh et al. (15) identified a Gq/AMPK/sphingolipid signaling pathway in macrophage–β cell communications. Selective activation of Gq-DREADD led to increased activation of AMPK, which remodeled the sphingolipid recycling pathways and led to release of sphingolipids and their derivatives from macrophages. CD36 on β cells facilitated the uptake of these lipids into β cells, leading to increased phosphorylation of PKCζ as well as reduced phosphorylation of AKT and GSK3β. The activation of GSK3β resulted in reduced insulin secretion. Singh et al. then identified GPR18 as an endogenous GPCR mediating this Gq-dependent pathway. (**B**) These findings position GPR18 as a potential target in T2D, alongside other GPCRs that have been implicated in insulin-regulating macrophage–β cell interactions.

## Version 1 11/16/2026

Electronic publication
